# Increases in atmospheric carbon dioxide: Anticipated negative effects on food quality

**DOI:** 10.1371/journal.pmed.1002600

**Published:** 2018-07-03

**Authors:** Kristie L. Ebi, Lewis H. Ziska

**Affiliations:** 1 Department of Global Health, University of Washington, Seattle, Washington, United States of America; 2 Adaptive Cropping Systems Laboratory, Agricultural Research Service, United States Department of Agriculture, Beltsville, Mississippi, United States of America

## Abstract

In a Perspective, Kristie Ebi and Lewis Ziska discuss Weyant and colleagues' accompanying study on the projected effects of atmospheric carbon dioxide on nutrition and disease.

## Summary points

- Higher atmospheric concentrations of carbon dioxide (CO_2_) increase the growth of cereal crops.
- At the same time, CO_2_ decreases the nutritional value of key staple crops, particularly rice and wheat, by lowering concentrations of protein, micronutrients, and B vitamins.
- From 2015–2050, elevated CO_2_ could result in an additional 125.8 million disability-adjusted life-years (95% credible interval [CrI] 113.6–138.9) globally, attributable to a greater burden of infectious diseases, diarrhea, and anemia.
- Impacts are projected to be greater in countries in the Southeast Asia and African regions.
- Implementing strategies to reduce greenhouse gas emissions could avert as much as 48.2% (95% CrI 47.8–48.5) of the health burden, compared with traditional public health interventions that could avert about 26.6% (95% CrI 23.8–29.6).

When increasing atmospheric concentrations of carbon dioxide (CO_2_) are discussed in the context of climate change, a silver lining is often postulated: “CO_2_ is plant food.” That is, higher concentrations of CO_2_ are generally acknowledged to stimulate plant photosynthesis and growth, with potential benefits for the productivity of the cereal crops that remain the world’s most important sources of food [1]. Cereal crops feed not just humans but also the animals that are important sources of protein for many. Since the mid-1960s, cereal production increased by approximately a billion tons [2], yet accelerated progress in agriculture is needed to keep pace with population growth anticipated to reach between 9–10 billion by 2050 [3], and to achieve Sustainable Development Goal (SDG) 2 to end hunger, achieve food security and improved nutrition, and promote sustainable agriculture.

But food security is about more than just production. While increases in CO_2_ may make some crops grow more quickly, research shows that higher CO_2_ concentrations can also reduce the nutritional quality of staple crops, from potatoes to barley, rice to wheat [4–6]. Understanding and describing the nature of these specific impacts is an important, if overlooked, aspect of climate change and food security, with obvious implications for early childhood and human development [7]. Documentation of the effects of CO_2_ on human nutrition has shown higher carbohydrate concentrations and reductions in plant-based protein and mineral content for many staple crops under experimental conditions [8].

So a key question remains: how will these documented changes in plants and agricultural products affect nutritional deficiencies globally? In an important study published in *PLOS Medicine*’s Special Issue on Climate Change and Health, Christopher Weyant and colleagues at Stanford University focus on two key micronutrients, zinc and iron, and report an analysis of climate change, dietary patterns, and disease risk across 137 countries [9]. They project that the effects of elevated atmospheric CO_2_ on zinc and iron concentrations could result in an additional 125.8 million disability-adjusted life-years (95% credible interval [CrI] 113.6–138.9) globally over the period 2015–2050, attributable to a greater burden of infectious diseases, diarrhea, and anemia. This increase is projected to have disproportionate effects in countries in the Southeast Asia and African regions, where populations already have high disease burdens associated with zinc and iron deficiencies.

Weyant and colleagues also begin to answer another fundamental issue: how projected changes in nutritional inequality might be avoided. They show that implementing strategies to reduce greenhouse gas emissions, such as those manifest in the Paris Agreement, surprisingly could avert as much as 48.2% (95% CrI 47.8–48.5) of this burden, compared with traditional public health interventions such as nutrient supplementation and disease control programs that could avert about 26.6% (95% CrI 23.8–29.6) of the burden.

Overall, Weyant and colleagues’ study highlights the magnitude of the challenges to health posed by increasing atmospheric CO_2_ concentrations and associated climate change with respect to global efforts to achieve SDG2. In addition, the authors show that traditional public health responses, such as supplementation and disease control, may be insufficient to the task.

The projected implications of this study for risks to health are large and significant in scope. But so too are the issues related to the effects of CO_2_ on plant chemistry and nutrition that have been less studied. For example, further research is needed to assess the possible effects of rising atmospheric CO_2_ on other plant-based compounds that have implications for human health, such as fatty acids, vitamins, or pharmacological compounds [6,10]. Yes, CO_2_ is plant food, but CO_2_-induced changes in plant chemistry will also have global consequences for all living creatures who consume plants, including humans. But the specific nature of those consequences will not be clear without additional research.

It should be noted that Weyant and colleagues focus only on the health consequences of increased CO_2_ concentrations and do not model how the climatic changes resulting from these emissions could affect all aspects of food security, including food access, utilization, and price stability, with subsequent consequences for undernutrition [11]. Increasing average temperatures and changing precipitation patterns are already reducing crop and food production in several world regions. Local temperature increases in excess of about 1°C above pre-industrial levels are projected to have further negative effects on yields for major crops (wheat and rice) in tropical and temperate regions. Furthermore, the negative effects of extreme weather and climate events, such as increases in the frequency, intensity, and duration of heatwaves, on production can result in rapid food and cereal price increases, reducing access to food, particularly among the poor. Together, increasing emissions of CO_2_ could decrease the quality, quantity, and availability of cereal crops important for the health of humans and, potentially, the animals that are important sources of protein.

Where to go from here? We need to elucidate the magnitude and pattern of the challenges to health and to identify and implement new evidence-based measures to reduce and/or manage risks. The range of unknowns is large: examples include understanding whether the CO_2_-induced decline in the nutritional value of food crops is linear and whether nutritional quality has already declined as a result of the increases in CO_2_ since the start of the industrial revolution. Investments are needed in health systems and technologies to improve nutrition, particularly among the most vulnerable people. We need investment in agriculture, particularly in identifying cultivars that may be less susceptible to nutritional deficits in a warming climate. Finally, and most importantly, as emphasized by the findings reported by Weyant and colleagues, to negate anticipated nutritional effects later in the century, we need large and rapid reductions in greenhouse gas emissions.

## Abbreviations

CO_2_: carbon dioxide
CrI: credible interval
SDG: Sustainable Development Goal
